# High-coverage, long-read sequencing of Han Chinese trio reference samples

**DOI:** 10.1038/s41597-019-0098-2

**Published:** 2019-06-14

**Authors:** Ying-Chih Wang, Nathan D. Olson, Gintaras Deikus, Hardik Shah, Aaron M. Wenger, Jonathan Trow, Chunlin Xiao, Stephen Sherry, Marc L. Salit, Justin M. Zook, Melissa Smith, Robert Sebra

**Affiliations:** 1 Department of Genetics and Genomic Sciences, Icahn School of Medicine at Mount Sinai, 1 Gustave L Levy Pl, New York, NY 10029 USA; 2 Material Measurement Laboratory, National Institute of Standards and Technology, 100 Bureau Dr, MS8312, Gaithersburg, MD 20899 USA; 3 Pacific Biosciences, 1305 O’Brien Drive, Menlo Park, CA 94025 USA; 4 National Center for Biotechnology Information, National Library of Medicine, National Institutes of Health, 45 Center Drive, Bethesda, MD 20894 USA; 5 Joint Initiative for Metrology in Biology, Stanford, CA 94305 USA; 6 Icahn Institute of Data Science and Genomic Technology, 1 Gustave L Levy Pl, New York, NY 10029 USA

**Keywords:** DNA sequencing, Genomics, Next-generation sequencing

## Abstract

Single-molecule long-read sequencing datasets were generated for a son-father-mother trio of Han Chinese descent that is part of the Genome in a Bottle (GIAB) consortium portfolio. The dataset was generated using the Pacific Biosciences Sequel System. The son and each parent were sequenced to an average coverage of 60 and 30, respectively, with N50 subread lengths between 16 and 18 kb. Raw reads and reads aligned to both the GRCh37 and GRCh38 are available at the NCBI GIAB ftp site (ftp://ftp-trace.ncbi.nlm.nih.gov/giab/ftp/data/ChineseTrio/). The GRCh38 aligned read data are archived in NCBI SRA (SRX4739017, SRX4739121, and SRX4739122). This dataset is available for anyone to develop and evaluate long-read bioinformatics methods.

## Abstract (summary)

**Table:** 

Design Type(s)	individual genetic characteristics comparison design • genotype design
Measurement Type(s)	whole genome sequencing assay
Technology Type(s)	DNA sequencing
Factor Type(s)	relationship • sex
Sample Characteristic(s)	Homo sapiens

Machine-accessible metadata file describing the reported data (ISA-Tab format)

## Background & Summary

Genome In a Bottle (GIAB) is a consortium hosted by the National Institute of Standards and Technology (NIST), primarily dedicated to the development and characterization of human genomic reference materials. The consortium includes representatives from government, industry, and academia. Currently, the GIAB portfolio includes seven genomes: the pilot genome NA12878 and two son-father-mother trios (one trio of Ashkenazi Jewish descent and the other of Han Chinese descent)^1^. The trio samples were selected from the Personal Genome Project with the aim of increasing reference sample diversity^2^. The GIAB genomes have been extensively sequenced on a number of different platforms)^1^. The datasets have been used to generate benchmark variant call sets for benchmarking and validating small variant calling methods^3,4^. The benchmark calls are based primarily on short-read data and cover approximately 90% of the human reference genome^3^. A number of medically relevant genes are difficult to characterize using short-read sequencing data^5,6^. Therefore, expanding the benchmark to more challenging variants and regions using long-read sequencing technologies is of interest to the consortium and its stakeholders, including technology and bioinformatics developers, clinical laboratories, and regulatory agencies^7^.

In an effort to expand the benchmark to more challenging variants and regions, a high-coverage long-read sequence dataset was generated for the Han Chinese Trio using the PacBio Sequel System (Pacific Biosciences, Menlo Park CA, USA). The Sequel System utilizes single molecule, real-time (SMRT) sequencing with fluorescently-labeled nucleotides^8^. In addition to being used to expand the benchmark set into more challenging variants and regions, the dataset will be used to improve phasing of variants and produce genome assemblies. This dataset can also be used by anyone to develop and evaluate long-read bioinformatics methods.

For the GIAB Han Chinese PacBio Sequel dataset, the son was sequenced to 60X coverage and parents to 30X coverage with a subread N50 of 16–18 kb. The raw reads and reads aligned to both the GRCh37 and GRCh38 are available at the NCBI GIAB ftp site (ftp://ftp-trace.ncbi.nlm.nih.gov/giab/ftp/data/ChineseTrio/). The GRCh38 aligned read data are archived in the NCBI Sequence Read Archive (SRA).

## Methods

### Experimental design

The Han Chinese GIAB trio (Table 1) samples were sequenced on the PacBio Sequel sequencing platform. The genomic DNA was used to prepare 14 sequencing libraries, 6 for the son and 4 each for the mother and father. 79 Sequel SMRT Cells were used to generate the dataset, with 46 SMRT Cells for the son, 17 for the father, and 16 for the mother. The subjects are part of the Personal Genome Project and provided informed consent for public availability of whole genome sequencing data and sample redistribution. The subjects are approved for “Public posting of personally identifying genetic information (PIGI)” by the Coriell and NIH/NIGMS IRBs. The study was approved by the NIST Human Subjects Protections Office and Coriell/NIGMS IRB.

**Table 1:** Sample names and identification numbers for GIAB Han Chinese trio. PGP ID - personal genome project identifier.

Sample	Coriell cell line ID	NIST ID	NIST RM #	NCBI BioSample	PGP ID
Chinese Son	GM24631	HG005	RM8393	SAMN03283350	hu91BD69
Chinese Father	GM24694	HG006	N/A^†^	SAMN03283348	huCA017E
Chinese Mother	GM24695	HG007	N/A^†^	SAMN03283349	hu38168C

^†^NIST Reference Materials are not planned for the Chinese parents, but cells and DNA are available from Coriell.

### Sample preparation

NIST RM8393 was used for HG005 sequencing libraries, and genomic DNA for HG006 and HG007 was obtained from Coriell (NA24694 and NA24695, respectively). Genomic DNA concentration was measured using the Qubit fluorimetry system with the High Sensitivity kit for detection of double-stranded DNA (Thermo Fisher, Part #Q32854). Fragment size distribution was assessed using the Agilent 2100 Bioanalyzer with the 12000 DNA kit (Agilent, Part 5067-1508). 20 µg high molecular weight genomic DNA was sheared using the Megaruptor instrument (Diagenode, Liege, Belgium) to 40 kb and the sheared DNA was used as input into the SMRTbell library preparation. SMRTbell libraries were prepared using the Pacific Biosciences Template Preparation Kit 1.0 - SPv3 (Pacific Biosciences, Part # 101-357-000). Once libraries were completed, they were size selected from 20–50 kb using the Blue Pippin instrument (Sage Science, Beverly MA, USA) to enrich for the longest insert lengths possible. The polymerase v2.0 binding kit (Part #101-862-200) was used to bind polymerase to SMRTbell templates. The binding complex was cleaned using the Column Clean-up kit (Pacific Biosciences, Part #100-184-100) before loading to remove excess polymerase and enhance loading efficiency.

### Pacific biosciences sequel system sequencing

SMRTbell libraries were sequenced on the Pacific Biosciences Sequel System using version 2 SMRT Cells (Part # 101-008-000) with 10-hour movies and diffusion loading at 6–7pM on plate. Two sequencing chemistries, Sequel Sequencing Kit 2.0 (Part # 101-053-000) and Sequel Sequencing Kit 2.1 (Part # 101-328-600) were used over the course of this project. For the son gDNA, kit 2.0 was used for 39 SMRT Cells and kit 2.1 for 3 SMRT Cells. For the parental gDNA, kit 2.1 was used for 21 SMRT Cells and kit 2.0 for 12 SMRT Cells. Individual SMRT Cell information including instrument used, date run, cell name (cell UUID), and cell lot is provided as Supplementary Tables 1–3.

### Sequence data processing

**Fig. 1:**
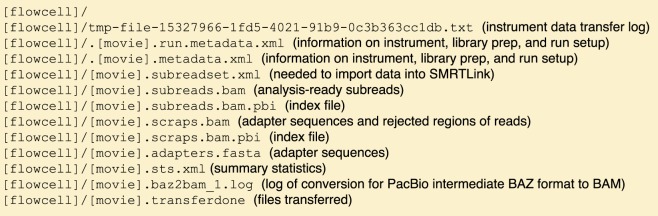
Raw data tar.gz directory structure.

Sequence data was exported from SMRT Link (version 5.0.1.9585) as tar.gz files using the “Export Data Sets” functionality. Each movie has one tar.gz file that contains sequence data in subreads BAM format and metadata (Fig. 1). FASTA files were extracted from subread BAMs using samtools (version 1.3.1, Li *et al*.^9^).

samtools fasta [movie].subreads.bam | gzip -c

Reads were aligned to reference genomes GRCh37 with hs37d5 decoy (ftp://ftp.1000genomes.ebi.ac.uk/vol1/ftp/technical/reference/phase2_reference_assembly_sequence/hs37d5.fa.gz) and GRCh38 with hs38d1 decoy (ftp://ftp.ncbi.nlm.nih.gov/genomes/all/GCA/000/001/405/GCA_000001405.15_GRCh38/seqs_for_alignment_pipelines.ucsc_ids/GCA_000001405.15_GRCh38_no_alt_plus_hs38d1_analysis_set.fna.gz). A representative subread per zero-mode waveguide (ZMW) was extracted with pbsv (pbsv version 2.0.0, https://github.com/PacificBiosciences/pbsv) and aligned to the reference with minimap2 (version 2.11-r797, Li^10^). Per-movie alignments were merged into a single aligned BAM and indexed using samtools (version 1.3.1).

pbsv fasta [movie].subreads.bam | \

minimap2 -t 8 -x map-pb -a –eqx -L -O 5,56 -E 4,1 -B 5 \

–secondary=no -z 400,50 -r 2k -Y [reference].fa - | \

samtools sort > [sample]_[movie]_[reference].bam

## Data Records

The GIAB Han Chinese trio genomes are available as EBV-immortalized cell lines and DNA from Coriell (Table 1). Genomic DNA from the son is available as a NIST Reference Material (RM8393). RM8393 genomic DNA was prepared from a single homogeneous culture by Coriell specifically for the NIST reference material.

The sequence data are available as raw data, sequences (FASTA), and aligned reads (BAM) at the NCBI GIAB ftp site (links below). The raw data are in the raw_data subdirectory as tar.gz files (Fig. 1). The tar.gz files are named using the following naming convention [Cell UUID].tar.gz. The compressed data archives include subreads as BAM files (BAM file format specifications http://samtools.github.io/hts-specs/SAMv1.pdf, PacBio BAM file format specifications https://pacbiofileformats.readthedocs.io/en/5.1/BAM.html). Sequence data are available in the PacBio_fasta subdirectory as gzipped FASTA files with the following naming convention [movie].subreads.fasta.gz. When base quality information is needed, e.g. read mapping, the subread BAM files in the raw_data can be used. The aligned read data are located in the PacBio_minimap2_bam subdirectory. The aligned reads are provided as BAM files along with their index (https://samtools.github.io/hts-specs/). The BAM file names use the following convention [NIST ID]_PacBio_[REF ID].bam, where [REF ID] indicates the reference genome that was used and is either GRCh37 or GRCh38. The GRCh38 aligned read data are archived in the NCBI Sequence Read Archive (SRA) under accessions NCBI SRA SRX4739017^11^ for HG005 Biosample SAMN03283350, SRX4739121^12^ for HG006 Biosample SAMN03283348, and SRX4739122^13^ for HG007 Biosample SAMN03283349. The three datasets are part of a larger GIAB project under SRP047086 with BioProject PRJNA200694. A list of FASTA files with the ftp paths for each sample can be obtained via a sequence index file (https://github.com/genome-in-a-bottle/giab_data_indexes/blob/master/ChineseTrio/sequence.index.ChineseTrio_NIST_MtSinai_PacBio_Sequel_fasta_09282018).

**Son** ftp://ftp-trace.ncbi.nlm.nih.gov/giab/ftp/data/ChineseTrio/HG005_NA24631_son/MtSinai_PacBio/

**Father** ftp://ftp-trace.ncbi.nlm.nih.gov/giab/ftp/data/ChineseTrio/HG006_NA24694-huCA017E_father/PacBio_MtSinai/

**Mother** ftp://ftp-trace.ncbi.nlm.nih.gov/giab/ftp/data/ChineseTrio/HG007_NA24695-hu38168_mother/PacBio_MtSinai/

## Technical Validation

**Fig. 2:**
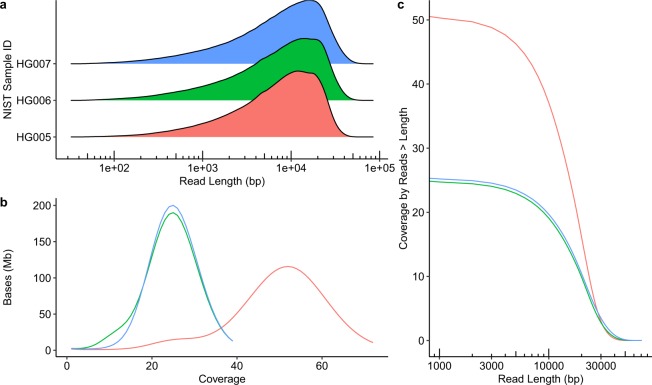
Read length and coverage for the three genomes. (**a)** Mapped read length distribution. (**b**) Number of genome positions (Mb - 10^6^ bases) by coverage. (**c**) Coverage by mapped read length.

The sequence dataset was characterized for number of reads, read length, coverage, mapping quality, and error rate. Mapped reads were used to characterize coverage, mapping quality, and error rate for the three samples. Metrics were calculated for reads mapped to GRCh37 using minimap2 (see Methods for details) using samtools stats. Nearly three times the number of SMRT Cells were used in sequencing HG005 compared to HG006 and HG007 (Table 2) resulting in approximately twice the total number of reads (Table 2). Improved loading efficiency was observed when using the later v2.1 sequencing chemistry. The majority (39/46) of SMRT Cells from HG005 were run with v2.0; whereas the majority (21/33) of SMRT Cells of the parental DNA was sequenced with v2.1. The polymerase did not change between v2.0 and v2.1 sequencing kits and therefore use of different sequencing kit is only expected to affect throughput and not error rates. Mean read length and N50 is similar across samples with mean subread lengths between 9.8 kb and 10.4 kb and N50 between 16.7 kb and 18.8 kb (Table 2, Fig. 2a). HG005 had approximately twice the coverage of HG006 and HG007 (Table 3, Fig. 2b). HG005 had ~15X coverage by reads >20 kb and HG006 and HG007 had ~10X coverage (Fig. 2c). The mapping rate was higher for HG005 compared to the other two samples (88% vs 83%). For HG006 the MQ0 rate (MQ0 rate is the percent of the mapped reads with a mapping quality of 0) was higher than the other two samples (0.40% versus 0.36% and 0.37%, Table 3). The base pair error rate is around 15% for all three samples.

**Table 2:** Pacific Biosciences Sequel run metrics.

		Samples		
		HG005	HG006	HG007
	SMRT Cells	46	17	16
Polymerase Reads	Reads (M)	18.4	9.0	8.9
	Avg. Length (kb)	11.0	11.0	11.3
	N50 (kb)	19.3	20.3	20.8
Subreads	Reads (M)	22.0	10.4	10.1
	Avg. Length (kb)	9.8	10.1	10.4
	N50 (kb)	16.7	18.3	18.8
Mapped Reads	Reads (M)	18.3	8.9	8.8
	Avg. Length (kb)	9.6	9.9	10.2
	N50 (kb)	16.3	17.9	18.3

Metrics are provided for polymerase reads, subreads, and mapped reads. Subreads (inserts) are sequences between SMRTbell adapters, the polymerase reads include SMRTbell adapters, and mapped reads are subreads mapped to GRCh37. Avg. Length (kb) - mean read length. Half of the sequenced bases are in reads longer than the N50.

**Table 3:** Read mapping summary metrics.

Sample	Coverage	Mapping Rate	MQ0 Rate	Error Rate
HG005	56.9	87.5%	0.37%	14.7%
HG006	28.5	82.9%	0.40%	14.9%
HG007	29.1	83.3%	0.36%	15.1%

Read mapping metrics were calculated for reads mapped to GRCh37 using minimap2. Coverage is the mean number of reads mapped to each position in the genome. Mapping rate is the number of mapped reads/ total number of subreads. MQ0 rate is the percent of the mapped reads with a mapping quality of 0 (i.e., reads that map equally well to multiple genomic locations). The error rate is the number of mismatches and gaps (insertions and deletions) in the alignment divided by the number of mapped bases. The number of mapped bases was calculated from the cigar string. Metrics were calculated from BAM files using the samtools stats command.

## Usage Notes

The data presented here can be used to evaluate different bioinformatic methods including small and structural variant calling, phasing, and genome assembly. All data from the Genome in a Bottle project are available without embargo, and the primary location for data access is ftp://ftp-trace.ncbi.nlm.nih.gov/giab/ftp. The data are also available as an Amazon Web Services Public Datasets repository with ‘s3://giab’ as bucket name and in the NCBI BioProject (http://www.ncbi.nlm.nih.gov/bioproject/200694). Additional information regarding data from the GIAB project can be obtained from GIAB github site (https://github.com/genome-in-a-bottle/). GIAB Analysis Team was formed to coordinate analyses. Analysis performed by the team are available on the ftp site (ftp://ftp-trace.ncbi.nlm.nih.gov/giab/ftp/data/ChineseTrio/analysis/). Analysis subdirectories generally use the following naming convention, [Dataset Name]_[Tool]_[Date (MMDDYYYY)]. Benchmark callsets are available at ftp://ftp-trace.ncbi.nlm.nih.gov/giab/ftp/release/ChineseTrio/ for use in evaluating small variant calling pipelines^3^. The Global Alliance for Genomics and Health Benchmarking Team published best practices for benchmarking germline small variant calls^4^. GIAB is actively developing structural variant benchmark sets and benchmarking methods. A draft structural variant benchmark set has been developed for another GIAB genome, HG002, is available and we plan to develop similar benchmark sets for the other GIAB genomes including the Chinese trio sequenced in this paper. For benchmarking structural variants we currently recommend Truvari (https://github.com/spiralgenetics/truvari) and SVanalyzer svbenchmark (https://svanalyzer.readthedocs.io/en/latest/), both of which are under active development. Future work is also planned to develop additional data and produce de novo assemblies and phased variants for these individuals, and GIAB welcomes community contributions of data and analyses.

## Supplementary Information

### ISA-Tab metadata file

Download metadata file

### Supplementary information

Supplementary Table 1

Supplementary Table 2

Supplementary Table 3
